# ^**111**^Indium Labelling of Recombinant Activated Coagulation Factor VII: In Vitro and Preliminary In Vivo Studies in Healthy Rats

**DOI:** 10.1155/2012/464810

**Published:** 2012-02-12

**Authors:** Amarnadh Nalla, Inge Buch, Maibritt Sigvardt, Rasmus Poul Bodholdt, Andreas Kjaer, Birger Hesse

**Affiliations:** ^1^Institute of Biomedical Sciences, Faculty of Health Sciences, University of Copenhagen, DK-2200, Copenhagen, Denmark; ^2^Faculty of Medical Laboratory Science, Metropolitan University College, DK-2200, Copenhagen, Denmark; ^3^Cluster for Molecular Imaging, Faculty of Health Sciences, University of Copenhagen, DK-2200, Copenhagen, Denmark; ^4^Department of Clinical Physiology, Nuclear Medicine & PET, Rigshospitalet, University of Copenhagen, DK-2100, Copenhagen, Denmark

## Abstract

The aim of this study is to investigate whether ^111^Indium-labelled recombinant FVIIa (rFVIIa) could be a potential radiopharmaceutical for localization of bleeding sources. DTPA-conjugated rFVIIa was radiolabelled with ^111^In chloride. In vitro binding efficiency of ^111^In-DTPA-rFVIIa to F1A2-Mab-sepharose was 99% in buffer, while it was 88–82% in serum. The binding efficiency of ^111^In-DTPA-rFVIIa to TF (1–209)-sepharose was 48% in buffer whereas 39%–36% in serum, respectively. In vivo experiment was conducted in healthy rats, and gamma camera images were taken immediately after iv. administration of 1.6–1.8 MBq ^111^In-DTPA-rFVIIa up to 120–130 min. Five min after administration of ^111^In-DTPA-rFVIIa, percentage of ^111^In activity was 6.0% in the cardiac region and 24.5% in the liver region. After 2 hours activity was decreased to 3.3% in heart while it had increased to 42.0% in the liver. The ^111^In-DTPA-rFVIIa might be a potential radiopharmaceutical for visualisation of tissues with significant TF expression such as acute bleeding lesions in the gastrointestinal tract.

## 1. Introduction

The aim of our investigation was to develop a new radiotracer using commercially available recombinant activated coagulation factor VII (rFVIIa, NovoSeven) for localisation of acute bleeding and cancer. rFVIIa is structurally and functionally similar to human plasma-derived coagulation factor VIIa, a glycoprotein of 406 amino acid residues with a molecular weight of 50 kDa [1]. It has been used in treatment of patients with various bleeding disorders like hemophilia A and B patients with inhibitory antibodies and patients with factor VII deficiency and Glanzmann thrombasthenia [2–7].

Blood clotting is initiated via tissue factor (TF), which is normally not exposed to the blood but is constitutively expressed on cells separated from the circulation. Vascular injury exposes TF-rich extravascular tissue, and FVIIa interacts with TF forming the FVIIa/TF complex and initiates the coagulation cascade. Through thrombin generation this leads to formation of a fibrin clot. In addition to its general haemostatic function, TF has also been reported to come in contact with FVIIa in the circulation as a result of structural defects in the vessel wall, angiogenic stimulation, entry to the bloodstream of large numbers of TF-expressing cells, which includes inflammatory leukocytes, leukaemic blast, and cancer cells [8–10]. Interaction between the FVIIa and TF induces the proliferation of certain cancer cells [11, 12]. Therapeutic agent conjugated to FVIIa or FVII resulted in specific targeted drug delivery of therapeutic agent to the TF-expressing tumor cells [13–16]. Several studies have shown overexpression and involvement of TF in rapidly growing malignant tumors [17].

TF has previously been targeted for imaging with antibody-coated perfluorocarbon emulsion nanoparticles against extracellular domain of TF (1–208) to identify the angioplasty-induced expression of tissue factor by smooth muscle cells within the tunica media in pigs [18]. In addition, fluorescence-conjugated antibodies against TF were used in real-time in vivo imaging of tissue factor during arterial thrombus formation in the mouse [19]. We suggest that rFVIIa could be a protein of potential interest for in vivo imaging by targeting to TF.

The localization of the rather frequently occurring acute gastrointestinal bleeding is often a serious clinical problem. Endoscopy and radiographic examinations including arteriography are the most commonly used imaging modalities for localization of bleeds, and treatment combined with endosonography is often successful [20, 21]. However, the lesion is not always localized by this, and explorative laparotomy comes into consideration as the final option. Even this very invasive approach, which is associated with a significant rate of complications, may occasionally also fail to identify the bleeding lesion, and other techniques including gamma camera imaging with ^99m^Tc-labelled red blood cells have been applied.

The latter technique is based on the principle of showing the extravasation site of the blood from the circulation, and bleeding scintigraphy has been quite useful [22]. The requirement for an excessive bleeding, that is, more than 1 mL/min [23, 24] at the time of tracer injection and subsequent image acquisition constitute is a serious limitation of the technique. Therefore, there is a need for a more sensitive, noninvasive diagnostic tool for localization of acute bleeding. We have previously hypothesized that rFVIIa could be potential tool to develop a radiotracer such as radiolabelled rFVIIa for localization of bleeding lesions [25] in order to visualize the bleeding site in a more direct manner, since rFVIIa binds directly to the tissue-exposing TF at the exact site of the bleeding lesion. Tc-99m would be the optimal radioisotope for radiolabelled rFVIIa, but earlier studies with such a tracer were unsuccessful [25]. ^111^In-labelled rFVIIa might be an alternative candidate for such a radiopharmaceutical.

The most common method attaching ^111^In to a protein is via a chelation reaction of a bifunctional chelating agent, which was previously conjugated to a protein or polypeptide. Diethylene-triaminepenta-acetic acid (DTPA) and its derivatives are the most popular chelating agents employed for ^111^In as well as some other metallic nuclides [26–28]. DTPA is often used as a bifunctional chelating agent conjugated to proteins like tumor-targeting probes such as monoclonal antibodies [29, 30] and Fab fragments [31, 32].The radiolabeling of proteins with trivalent metallic isotopes and ability of DTPA to react with proteins to establish a stable amide bonds in a single-step process are well documented [26, 33–35], and ^111^Indium labeling can be performed without loss of labelling efficiency after long-term storage DTPA-conjugated protein [36].

## 2. Materials and Methods

### 2.1. Materials

We used ^111^In chloride from Tyco Healthcare/Mallinckrodt Medical, cyclic diethylene triamine pentaacetic acid (cDTPA), dimethyl sulfoxide (DMSO) anhydrous, trisodium citrate dehydrate, glycyl-glycine, Costar Spin-X centrifuge tube from Sigma-Aldrich, and ITLC-SG from Pall Corporation, USA. rFVIIa, TF (1–209) and monoclonal mouse anti-FVII antibody, and F1A2 were from Novo Nordisk A/S, Bagsværd, Denmark. Slide-A-Lyzer Dialysis Cassettes were from Thermo Fisher Scientific, IL, USA. CNBr-activated sepharose-4B and PD-10 columns were obtained from Pharmacia, Uppsala, Sweden. All other reagents used in the experiments were analytical-grade products from Sigma-Aldrich if not mentioned from other suppliers.

### 2.2. Preparation of DTPA-rFVIIa

rFVIIa in glycyl-glycine buffer, pH 7.5 (10 mM glycyl-glycine, 150 mM NaCl, 10 mM CaCl_2_) was dialysed overnight against 2 times 2.5 litres of borate buffer saline pH 8.2 containing 10 mM CaCl_2_. cDTPA (1 : 1 ratio of DTPA to rFVIIa) dissolved in DMSO anhydrate was added to the 1.35 mg/mL rFVIIa and incubated at +4°C for 4 hours. After the incubation, free DTPA was removed by overnight dialysis against 2 times 2 litres of glycyl-glycine buffer, pH 7.4. The DTPA-rFVIIa was aliquoted and stored at −80°C for radiolabeling with ^111^In-chloride.

### 2.3. Radiolabeling

The radiolabeling was carried out by addition of double volume of 500 mmol/l sodium citrate buffer, pH 6 to 10 Mbq of ^111^In chloride (2 : 1 v/v; buffer: ^111^In chloride). After 5 min 20 *μ*L of DTPA-rFVIIa conjugate prethawed at RT was added, and the reaction was mixed and then incubated at RT for 30 min. For the rat experiments, 100 *μ*L of DTPA-rFVIIa labelled with 37 MBq of ^111^In chloride was used. Finally ^111^In-labelled DTPA-rFVIIa was purified using glycyl-glycine buffer-equilibrated PD-10 column and eluted with glycyl-glycine buffer.

### 2.4. Labelling Efficiency

Labelling efficiency was determined on ITLC-silica-impregnated glass fiber sheets (ITLC-SG) from the final product, and 5 *μ*L was applied to the ITLC strip that was developed with 50 mmol/l sodium citrate buffer for 5–10 min. Radioactivity was measured by an instant chromatography scanner (VCS-101, Veenstra Instruments) equipped with a NaI crystal.

### 2.5. Immobilization of TF (1–209) and F1A2-Mab

The soluble TF (1–209) and Ca^2+^-dependent monoclonal mouse anti-FVII antibody, F1A2-MAB, were immobilised on to CNBr-activated sepharose-4B with TF (1–209) or with F1A2-MAB as described by the manufacturer at a final density of 9 mg TF (1–209) per mL sepharose-4B and 10 mg F1A2-MAB per mL sepharose-4B. Finally TF-(1–209) and F1A2-MAB-immobilised sepharose-4B was blocked with 1 M glycine, 0.1 M NaHCO_3_, 0.5 M NaCl, pH 8.5.

### 2.6. ^111^In-Labelling, Serum Stability and Binding Efficiency Studies

Stability studies on ^111^In-labelled DTPA-rFVIIa were performed by addition of 100 *μ*L of 100 mM EDTA and incubated for 5 min after radiolabelling in citrate buffer for 30 min. Then labelling efficiency was determined as mentioned earlier. Stability studies were performed in assay buffer (10 mM glycyl-glycine, 150 mM NaCl, 10 mM CaCl_2_, 0.1% BSA, pH 7.5) and in human serum with ^111^In-DTPA-rFVIIa from three different radiolabeling. A 5–10 *μ*L of 2.5 *μ*g ^111^In-DTPA-rFVIIa elute from PD-10 column was added to 270 *μ*L human serum and incubated for 1 hour with constant shaking at room temperature. After the incubation 100 *μ*L TF (1–209)-sepharose or F1A2-MAB-sepharose (50% suspension) was added, and the binding was allowed for 1 hour at room temperature with constant shaking. Stability studies in assay buffer and serum for 0 hour were performed similarly to 1-hour serum incubation studies without prior incubation for 1 hour in assay buffer or serum. Total reaction content was centrifuged in Costar Spin-X centrifuge cellulose acetate membrane filter tubes with pore size of 0.45 *μ*m at 6000 rpm for 10 min using Heraues Biofuge 15, Bie & Berntsen, Denmark. The radioactivity of sepharose beads and filtrate was measured using Cobra II auto-gamma, Packard.

The binding efficiency of ^111^In-DTPA-rFVIIa to TF (1–209)-sepharose and F1A1-MAB-sepharose was calculated using the following formula:

(1) $$\begin{array}{cc} & \text{Binding}\;\text{efficiency}\;\left(\%\right)\\ & \;\;=\frac{\text{Sepharose}\;\text{beads}\;\left(\text{cpm}\right)-\text{Filterate}\;\left(\text{cpm}\right)}{\text{Sepharose}\;\text{beads}\;\left(\text{cpm}\right)+\text{Filterate}\;\left(\text{cpm}\right)-2*\text{Background}\;\left(\text{cpm}\right)}*100\%. \end{array}$$

### 2.7. In Vivo Studies in Rats

Animal care and all experimental procedures were performed under the approval of the Danish Animal Welfare Council (2006/561-1124). An iv. cannula was introduced into a tail vein in 6 healthy, male Sprague-Dawley rats, mean bwt 303 g (range 284–323), three rats at a time with 30-min interval. They were anaesthetized with a mixture of Hypnorm (fentanyl citrate 0.315 mg/mL and fluanisone 10 mg/mL) and Dormicum (midazolam 5 mg/mL), 0.3 mL/100 g body weight, and maintained anaesthetized with 0.15 mL/100 g body weight every 20 min. 1.6–1.8 MBq ^111^In-DTPA-rFVIIa was injected into the cannula and flushed with 0.3 mL saline solution. Immediately following the injection the group of three rats at a time were positioned on a Mediso 1-headed gamma camera (Mediso Medical Imaging Systems, Budapest, Hungary) with a medium energy, general purpose collimator. Six dynamic 5-min images were acquired immediately after radioisotope injection, followed by static images after 1 and 2 hours. After the end of the two hours image acquisition of the last three rats 70 MBq of ^99m^Tc-labelled human serum albumin (Vasculosis) was injected into the tail vein of rats 4–6 to verify the regions of interest (ROI) definitions of the heart and the liver.

Dynamic and static images were obtained in a matrix size of 256 × 256. ROIs were drawn around the heart, the liver, and the total body, which were rather easy to define in all rats from the indium images and in agreement with the ^99 m^Tc-labelled albumin images created for definitions of the ROIs. Indium count rates were not corrected for physical decay or background. After the last image acquisition rats were sacrificed, and a sample of around 1 mL blood was successfully obtained from the neck vessels, and count rates (cps/mL anticoagulated blood) were determined in a COBRA well counter. Assuming a mean total blood volume in each rat of 20 mL (corresponding to a human blood volume of 5 litres/70 kg bwt), the fraction of the injected dose of ^111^In activity still circulating in the vascular system was calculated by comparison with the count rate of an ^111^In standard determined in the COBRA well counter.

## 3. Results

### 3.1. ^111^In Labelling, Stability, and Binding Efficiencies

**Figure 1:**
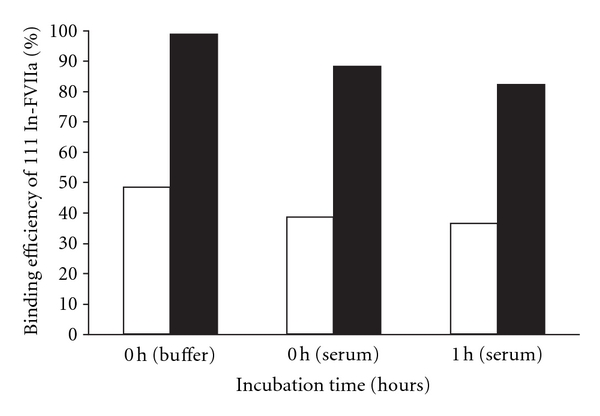
In vitro binding efficiency of ^111^In-FVIIa to TF (1–209)-sepharose and F1A2-MAB-sepharose: Median values of 3 individual experiments on binding efficiency of ^111^In-FVIIa to TF (1–209)-sepharose (empty bars) and F1A2-MAB-sepharose (solid bars) in assay buffer and human serum at 0-hour and after 1-hour incubation.

The radiolabeling yield of the purified product of ^111^In-DTPA-rFVIIa varied between 88–97%, and specific activities of 9–13 MBq/*μ*mol were used for in vitro studies. No significant loss of ^111^Indium bound to DTPA-rFVIIa was observed even after addition of the strong chelator like EDTA. The median binding efficiency of ^111^In-DTPA-rFVIIa to F1A2-Mab-sepharose was 99% (84–99%) in assay buffer, and it was 88% (87-88%) just after the labelling and fell to 82% (range 79–85%) after 1-hour incubation in the serum stability study. The binding efficiency of ^111^In-DTPA-rFVIIa to TF (1–209)-sepharose was 39% (range 28–44%) for 0 hour and decreased to 36% (range 33–48%) after 1 hour in serum compared to 48% (range 41–53%) after 1-hour incubation in assay buffer (Figure 1).

### 3.2. In Vivo

**Figure 2:**
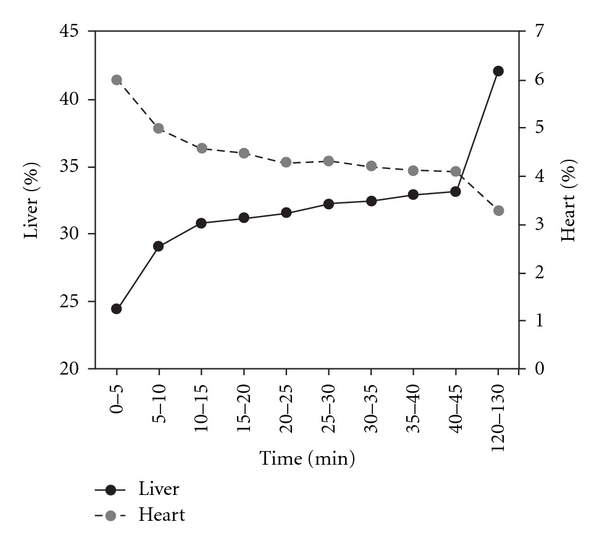
In vivo ^111^In-FVIIa activity in liver and heart versus total radioactivity: changes over time of ^111^In radioactivity (median values) in 6 rats in two regions of interest corresponding to the heart (grey circles) and liver (black circles) against total.

**Figure 3:**
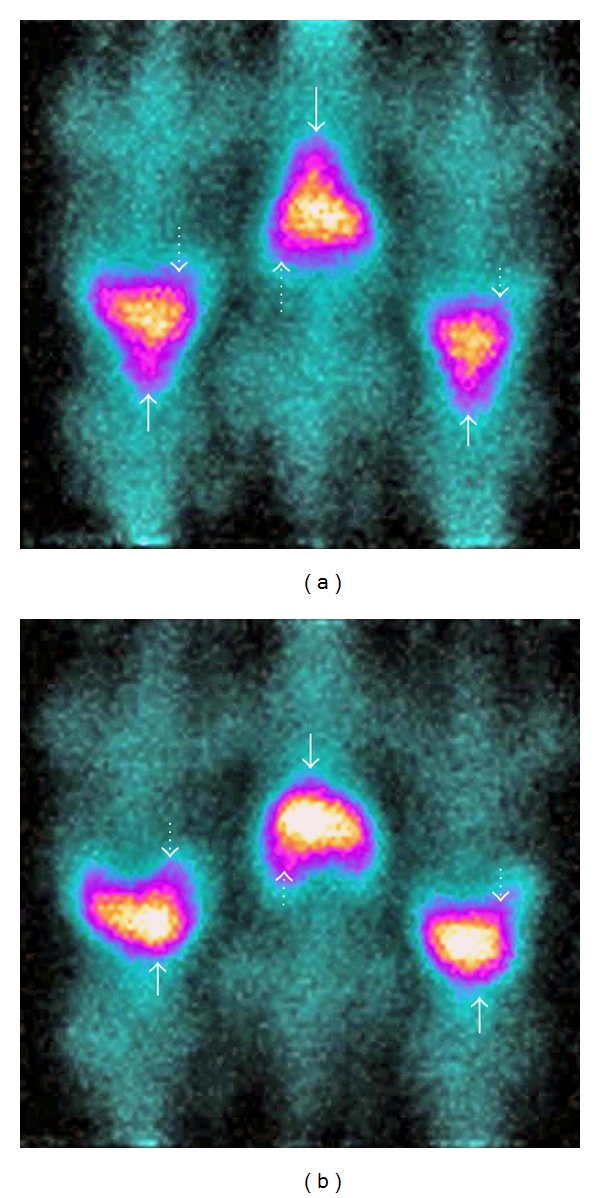
^111^In-FVIIa scintigraphy in three rats with early and delayed images: scintigraphy of three rats at 1–10 min (a) and 2 hours (b) after iv. injection of 1.6–1.8 MBq ^111^In-FVIIa. The pointed arrows show the splanchnic organs; filled arrows point to the cardiac region.

After injection of rFVIIa into the rats, heart and big blood vessels were immediately visualized. The splanchnic organs were also clearly identified in the early images and slowly increased their activity uptake. An ROI around the heart in the six rats during the first 5 minutes showed a median value of 6.0%  ^111^In activity, calculated as the count rate in the cardiac region as percentage of the total rat count rate. It was 24.5% in the splanchnic (liver) region in percentage of total rat count rate; over the following two hours it gradually decreased to a median 3.3% (range 2.7–4.5%), that is, to about half the initial value and increased to 42.0% in the splanchnic region (Figures 2 and 3). Assuming that the heart contains around 5% of the total rat blood volume (in man around 250/5000 mL), it can be estimated that after 5 minutes the In-rFVIIa circulating activity is ca. 20 × 5%; that is, all In-rFVIIa activity is within the circulation. After 10 minutes it had decreased to ca. 80% of total In-rFVIIa activity. After 2 hours this value was reduced to about 50%. The blood samples showed similar orders of magnitude. The average value contained 48 kBq (40–55 kBq), that is, around 40% of the injected activity of 1.5 MBq.

## 4. Discussion

Current methods for the detection of lesions causing acute gastrointestinal bleeding have significant limitations of sensitivity, including scintigraphy with ^99 m^Tc-labelled autologous red blood cells, which often requires a rather high rate of active bleeding; imaging of radiolabelled rFVIIa could provide a potentially more sensitive, noninvasive diagnostic tool. Factor VII is a very important component in the coagulation cascade. It is present in the circulating blood and binds to TF when exposed at the site of injury. The radioisotope of choice for gamma camera imaging would of course be ^99 m^Tc, but unfortunately in our recent study we failed to create a ^99 m^Tc-labelled rFVIIa ligand stable in vivo [25], ^111^In could be an alternative radioisotope as demonstrated, for example, with the very successful tracer for somatostatin receptor imaging, ^111^In-labelled Pentreotide [37]. This preliminary study has shown that rFVIIa can be effectively labeled with ^111^In with preserved biological properties.

It is always a challenge to develop a radiopharmaceutical without loss of intact biological activity. A minor loss of intact biological activity during the radiolabeling may almost always be anticipated because of the influence of even subtle changes related to the radiolabeling procedure on the molecular structure, which determines the biomolecular property. In order not to affect the biological activity of rFVIIa and to avoid the protein cross-linking associated with high DTPA to ligand ratio [35], we reduced the DTPA : rFVIIa ratio to a value as low as possible (1 : 1). The DTPA conjugation was performed in borate saline buffer with pH 8.2 containing 10 mM CaCl_2_, which is a good buffer for the conjugation; however, high pH may affect the stability of rFVIIa. ^111^Indium labelling of DTPA-rFVIIa was performed in citrate buffer, which among different buffers tested gave the highest labelling efficiency. But the citrate buffer may be less optimal because of its chelating properties of calcium ions, the presence of which is essential for the preservation of biological activity of rFVIIa. The conjugation and labelling conditions were sub-optimal, but with compromises we obtained a labelling yield of rFVIIa which was in an acceptable range. Unstable and nonspecific binding of indium to DTPA-protein conjugate may lead to increased liver uptake and higher background activity, previously reported [38]. To avoid this problem and to demonstrate stability of ^111^In-DTPA-rFVIIa complex, we introduced EDTA to the labelling procedure prior to serum stability studies. Analysis of conjugation efficiency and protein cross-linking needs to be considered in future studies with conjugation of rFVIIa with cDTPA, which might reveal loss of in vitro binding efficiency to sTF (1–209).

The binding efficiency of ^111^In-DTPA-rFVIIa to TF (1–209) was reduced compared to F1A1-MAB. This may suggest that rFVIIa sites involved in the binding to TF are more sensitive to the labelling procedure than the epitope for the F1A2 antibody. The observed loss of biological activity may be due to an elevated pH during the conjugation, lack of calcium ions during the radiolabeling, the conjugation of DTPA at the binding site of TF (1–209), interchain cross-linking of rFVIIa by DTPA, and finally by oxidation of methionine residues of rFVIIa [35, 39, 40], which may lead to reduction of the binding efficiency of ^111^In-DTPA-rFVIIa to TF (1–209). Modification of the conditions for conjugation and for the radiolabeling procedure could improve the binding efficiency of ^111^In-DTPA-rFVIIa especially to TF (1–209) in vitro. Our results suggest that the N-terminal gamma glutamic acid domain, which is a calcium binding domain of rFVIIa unaffected by the DTPA conjugation and by ^111^In-labelling whereas it interfered to some degree with the TF binding domain. The binding of rFVIIa to TF is a crucial step to form the FVIIa/TF complex and to initiate the coagulation cascade through generation of thrombin and formation of a stable fibrin clot. In vitro serum stability studies of ^111^In-DTPA-rFVIIa were performed in human serum to examine the risk of human serum-induced enzymatic stability. ^111^In-DTPA-rFVIIa was stable in serum for 1 hour with a negligible loss of its binding efficiency to TF (1–209) and F1A2-MAB compared to the binding efficiency in assay buffer. 

In vivo studies performed in rats suggest that all ^111^In-DTPA-rFVIIa activity was circulating after 5 minutes. After 10 minutes the ^111^In-activity had decreased to around 80% and after 2 hours to around 50% according to the calculation of ^111^In-activity in the cardiac ROI of the gamma camera images, that is, a ROI almost only representing blood volume activity (^99m^Tc-labelled human serum albumin images were used for confirmation of ROI). The fairly consistent two-hour values calculated from the gamma camera images were in nice agreement with the calculation from the blood samples drawn after two hours. The data thus strongly suggest that 40–50% of the injected ^111^In activity is circulating after two hours, thus allowing for a longer time span for possible imaging than the current radionuclide technique using ^99m^Tc-labelled red cells.

The circulating half-life of ^111^In-DTPA-rFVIIa was measured both by using calculations of in vivo imaging data and by counting blood samples as an in vitro measurement. In vivo images from the six rats give complete biodistribution, and they were very consistent therefore we find it is not necessary to do radioactivity measurement in each major organ. It must be added that we only measured ^111^In acitivity in the images and blood samples. Theoretically those count rate data could represent other ^111^In compounds or free ^111^indium, but the latter seems unlikely, since free ^111^indium is rapidly excreted via the kidneys, which never got visible in our images. Other In-labeled compounds from rFVIIa metabolism cannot be excluded, but appear less likely, since rFVIIa is primarily excreted via the liver and does not remain in the circulation.

The in vivo imaging analysis was performed for 2 hours due to the half-life of circulating rFVIIa reported to be around 2-3 hours with some variations in pharmacokinetic studies in rodents. Since the half-life for ^125^I-rFVIIa, considered to closely reflect biological rFVIIa activity, in rats is 80–90 min [41], we restricted our imaging time for a similar duration. An optimal time window for in vivo imaging of acute bleeding would ideally allow for imaging up to several hours after tracer injection, because acute gastro-intestinal bleeding often occurs as repeated bleeding periods for a few hours interrupted by hours of no bleeding. Such a time window demands a tracer with a longer half-life in the circulation. In this study, investigation of ^111^In-DTPA-rFVIIa, performed in healthy rats only, serves as a model and a first step to show whether it is potentially useful to apply ^111^In-DTPA-rFVIIa for imaging acute bleeding lesions and possible other disease. This has to be shown in future investigations.

Since significant TF expression has also been found in several cancers [42] and apparently related to formation of distant metastases [43–46], a radiolabelled gamma emitter or preferably a positron emitter-labeled rFVIIa could be interesting for the diagnosis of or monitoring of progression in some malignant diseases. The time interval for cancer imaging may need to be longer, allowing for the binding of the ligand to less well-perfused tumour cells and clearing of surrounding tissue. Thus ^111^In-labelled rFVIIa or possibly rather TF with active site-inhibited FVIIa (ASIS) could be suitable also for cancer imaging [13]. PET isotopes like ^68^Ga (half-life: 68 min) or ^64^Cu (half-life: 12.7 h), labelled to DOTA-conjugated rFVIIa, may be a potentially better isotope for cancer imaging with the advantage of PET/CT with significantly better spatial image resolution, a higher sensitivity, and being quantitative.

**Limitations of the Study**

The in vivo study included six rats only, which is a very limited number of experimental animals, and the animals only comprised healthy rats without acute bleeding. However, the values were very consistent in the six rats, and in a previous study in bleeding rabbits it has been shown that ^123^I-rFVIIa can detect acute gastrointestinal bleeding [47], but unfortunately ^123^I is not useful for acute imaging purposes because of its shorter half-life.

## 5. Conclusion

Recombinant FVIIa can be labelled successfully with ^111^Indium chloride; ^111^In-DTPA-rFVIIa is quite stable in vitro, also incubated in serum, with a minor loss of binding efficiency to TF (1–209) and F1A2-MAB compared to binding efficiency in assay buffer. Half of the ^111^In-labelled rFVIIa was still in circulation two hours after iv. injection. Thus it may be a potential radiopharmaceutical for visualisation of tissues with significant TF expression such as acute bleeding lesions in the gastrointestinal human tract and possibly also for cancer imaging.

## Abbreviations

cDTPA: Cyclic diethylene-triaminepenta-acetic acid
DTPA: Diethylene-triaminepenta-acetic acid
F1A2-MAB: Anti-FVII monoclonal antibody
ITLC: Instant thin-layer chromatography
rFVIIa: Recombinant activated factor VII
TF (1–209): Tissue factor (residues 1–209)
TF: Tissue factor
ROI: Region of interest
RT: Room temperature
BWT: Body weight.
